# Supplementary material for: RNA exosome mutations in pontocerebellar hypoplasia alter ribosome biogenesis and p53 levels
Source: Life Sci Alliance. 2020 Jun 11;3(8):e202000678. doi: 10.26508/lsa.202000678 (PMC7295610; doi:10.26508/lsa.202000678)
Supplement: Supplementary file 2 [file LSA-2020-00678_TableS2.docx]

| **Gene ID** | **Gene name** | **Ortholog** | **Panther Family** | **Panther Protein class** |
| --- | --- | --- | --- | --- |
| DANRE\|ZFIN=ZDB-GENE-030131-9830\|UniProtKB=B0S6A9 | wdr3 | WD repeat domain 3;wdr3;ortholog | WD REPEAT-CONTAINING PROTEIN 3 (PTHR19853:SF0) | small subunit biogenesis |
| DANRE\|ZFIN=ZDB-GENE-030131-4917\|UniProtKB=B8JKQ1 | esf1 | ESF1, nucleolar pre-rRNA-processing protein, homolog (S. cerevisiae);esf1;ortholog | ESF1 HOMOLOG (PTHR12202:SF0) |  |
| DANRE\|ZFIN=ZDB-GENE-050417-261\|UniProtKB=F1R9Z2 | cebpz | CCAAT/enhancer-binding protein (C/EBP), zeta;cebpz;ortholog | CCAAT/ENHANCER-BINDING PROTEIN ZETA (PTHR12048:SF0) | transcription factor(PC00218) |
| DANRE\|ZFIN=ZDB-GENE-030131-9685\|UniProtKB=F1QLW8 | ddx18 | RNA helicase;ddx18;ortholog | ATP-DEPENDENT RNA HELICASE DDX18 (PTHR24031:SF301) | large subunit biogenesis |
| DANRE\|Ensembl=ENSDARG00000098507\|UniProtKB=A0A0G2L833 | lyar | Ly1 antibody reactive homolog (mouse);lyar;ortholog | CELL GROWTH-REGULATING NUCLEOLAR PROTEIN (PTHR13100:SF10) |  |
| DANRE\|ZFIN=ZDB-GENE-040927-28\|UniProtKB=Q6DRI7 | ddx51 | ATP-dependent RNA helicase DDX51;ddx51;ortholog | ATP-DEPENDENT RNA HELICASE DDX51 (PTHR24031:SF68) |  |
| DANRE\|ZFIN=ZDB-GENE-040912-163\|UniProtKB=Q66I02 | utp3 | UTP3, small subunit (SSU) processome component, homolog (S. cerevisiae);utp3;ortholog | SOMETHING ABOUT SILENCING PROTEIN 10 (PTHR13237:SF8) | RNA binding protein(PC00031), small subunit biogenesis |
| DANRE\|ZFIN=ZDB-GENE-050522-321\|UniProtKB=F1QDT3 | nle1 | Notchless homolog 1 (Drosophila);nle1;ortholog | NOTCHLESS PROTEIN HOMOLOG 1 (PTHR19848:SF0) | large subunit biogenesis |
| DANRE\|ZFIN=ZDB-GENE-050522-359\|UniProtKB=A0A1L1QZC5 | ddx47 | DEAD (Asp-Glu-Ala-Asp) box polypeptide 47;ddx47;ortholog | ATP-DEPENDENT RNA HELICASE DDX47-RELATED (PTHR24031:SF78) |  |
| DANRE\|ZFIN=ZDB-GENE-030131-9345\|UniProtKB=Q7SX91 | rrs1 | Ribosome biogenesis regulatory protein;rrs1;ortholog | RIBOSOME BIOGENESIS REGULATORY PROTEIN HOMOLOG (PTHR17602:SF4) | ribosomal protein(PC00202), large and small subunit biogenesis |
| DANRE\|ZFIN=ZDB-GENE-030131-9837\|UniProtKB=A0A140LG68 | rrp1 | Ribosomal RNA-processing 1;rrp1;ortholog | RIBOSOMAL RNA-PROCESSING 1 (PTHR13026:SF0) |  |
| DANRE\|ZFIN=ZDB-GENE-080303-28\|UniProtKB=F1Q829 | mrpl22 | Mitochondrial ribosomal protein L22;mrpl22;ortholog | 39S RIBOSOMAL PROTEIN L22, MITOCHONDRIAL (PTHR13501:SF8) | ribosomal protein(PC00202) |
| DANRE\|ZFIN=ZDB-GENE-030616-5\|UniProtKB=Q7ZVR1 | wdr75 | WD repeat-containing protein 75;wdr75;ortholog | WD REPEAT-CONTAINING PROTEIN 75 (PTHR44215:SF1) |  |
| DANRE\|ZFIN=ZDB-GENE-030131-2621\|UniProtKB=B0V0U5 | urb2 | URB2 ribosome biogenesis 2 homolog (S. cerevisiae);urb2;ortholog | UNHEALTHY RIBOSOME BIOGENESIS PROTEIN 2 HOMOLOG (PTHR15682:SF2) |  |
| DANRE\|ZFIN=ZDB-GENE-110411-38\|UniProtKB=F8W272 | znhit6 | Zinc finger HIT-type-containing 6;znhit6;ortholog | BOX C/D SNORNA PROTEIN 1 (PTHR13483:SF3) | large subunit biogenesis |
| DANRE\|ZFIN=ZDB-GENE-031118-110\|UniProtKB=Q6ZM19 | eif6 | Eukaryotic translation initiation factor 6;eif6;ortholog | EUKARYOTIC TRANSLATION INITIATION FACTOR 6 (PTHR10784:SF0) | translation initiation factor(PC00224), large subunit biogenesis |
| DANRE\|ZFIN=ZDB-GENE-030131-9670\|UniProtKB=Q7ZV23 | snu13b | NHP2 non-histone chromosome protein 2-like 1 (S. cerevisiae);snu13b;ortholog | NHP2-LIKE PROTEIN 1 (PTHR23105:SF38) | ribosomal protein(PC00202), large and small subunit biogenesis |
| DANRE\|ZFIN=ZDB-GENE-030131-6989\|UniProtKB=Q6DRD0 | ebna1bp2 | EBNA1 binding protein 2-like;ebna1bp2;ortholog | RRNA-PROCESSING PROTEIN EBP2-RELATED (PTHR13028:SF0) | large subunit biogenesis |
| DANRE\|ZFIN=ZDB-GENE-030114-4\|UniProtKB=F1QUZ5 | ppan | Peter pan homolog (Drosophila);ppan;ortholog | HCG2039996-RELATED (PTHR12661:SF5) | large subunit biogenesis |
| DANRE\|ZFIN=ZDB-GENE-030131-464\|UniProtKB=A3KPR1 | wdr36 | WD repeat domain 36;wdr36;ortholog | WD REPEAT-CONTAINING PROTEIN 36 (PTHR22840:SF12) |  |
| DANRE\|ZFIN=ZDB-GENE-020419-35\|UniProtKB=Q6NYD4 | mak16 | Protein MAK16 homolog;mak16;ortholog | PROTEIN MAK16 HOMOLOG (PTHR23405:SF4) | large subunit biogenesis |
| DANRE\|ZFIN=ZDB-GENE-040426-2117\|UniProtKB=Q7SXA1 | CU469526 | Ribosomal protein L26;rpl26;ortholog | RIBOSOMAL PROTEIN L26 (PTHR11143:SF7) | ribosomal protein(PC00202), large subunit biogenesis |
| DANRE\|ZFIN=ZDB-GENE-050309-7\|UniProtKB=F1QWP7 | nop2 | NOP2 nucleolar protein homolog (yeast);nop2;ortholog | 28S RRNA (CYTOSINE(4447)-C(5))-METHYLTRANSFERASE-RELATED (PTHR22807:SF30) | large subunit biogenesis |
| DANRE\|ZFIN=ZDB-GENE-021231-3\|UniProtKB=A9JR96 | wdr43 | WD repeat domain 43;wdr43;ortholog | WD REPEAT-CONTAINING PROTEIN 43 (PTHR44267:SF1) |  |
| DANRE\|ZFIN=ZDB-GENE-060825-113\|UniProtKB=Q1LWH6 | fam207a | Family with sequence similarity 207, member A;fam207a;ortholog | PROTEIN FAM207A (PTHR31109:SF2) | small subunit biogenesis |
| DANRE\|ZFIN=ZDB-GENE-040426-2501\|UniProtKB=Q6P0C5 | rpf2 | Brix domain containing 1;rpf2;ortholog | RIBOSOME PRODUCTION FACTOR 2 HOMOLOG (PTHR12728:SF0) | nuclease(PC00170), large subunit biogenesis |
| DANRE\|ZFIN=ZDB-GENE-030131-2146\|UniProtKB=E7F5L8 | mphosph10 | U3 small nucleolar ribonucleoprotein protein MPP10;mphosph10;ortholog | U3 SMALL NUCLEOLAR RIBONUCLEOPROTEIN PROTEIN MPP10 (PTHR17039:SF0) | ribonucleoprotein(PC00201) |
| DANRE\|Ensembl=ENSDARG00000098859\|UniProtKB=A0A0G2KTX1 | CABZ01079760 | Uncharacterized protein;unassigned;ortholog | NUCLEOLAR PROTEIN 14 (PTHR23183:SF0) | ribosomal protein(PC00202), small subunit biogenesis |
| DANRE\|ZFIN=ZDB-GENE-041007-4\|UniProtKB=Q6DRH5 | nop10 | H/ACA ribonucleoprotein complex subunit 3;nop10;ortholog | H/ACA RIBONUCLEOPROTEIN COMPLEX SUBUNIT 3 (PTHR13305:SF0) | ribosomal protein(PC00202) |
| DANRE\|ZFIN=ZDB-GENE-030131-616\|UniProtKB=Q6DRP2 | gnl3 | Guanine nucleotide-binding protein-like 3;gnl3;ortholog | GUANINE NUCLEOTIDE-BINDING PROTEIN-LIKE 3 (PTHR11089:SF11) |  |
| DANRE\|ZFIN=ZDB-GENE-040426-2466\|UniProtKB=A0A0R4IJ17 | utp4 | Cirrhosis, autosomal recessive 1A (cirhin);utp4;ortholog | U3 SMALL NUCLEOLAR RNA-ASSOCIATED PROTEIN 4 HOMOLOG (PTHR44163:SF1) | small subunit biogenesis |
| DANRE\|ZFIN=ZDB-GENE-030131-8414\|UniProtKB=Q6TNS2 | pak1ip1 | p21-activated protein kinase-interacting protein 1-like;pak1ip1;ortholog | P21-ACTIVATED PROTEIN KINASE-INTERACTING PROTEIN 1 (PTHR42968:SF32) | large subunit biogenesis |
| DANRE\|ZFIN=ZDB-GENE-031118-120\|UniProtKB=F1Q749 | dkc1 | Dyskeratosis congenita 1, dyskerin;dkc1;ortholog | H/ACA RIBONUCLEOPROTEIN COMPLEX SUBUNIT 4 (PTHR23127:SF0) | centromere DNA-binding protein(PC00071) |
| DANRE\|ZFIN=ZDB-GENE-021031-3\|UniProtKB=A0A0R4IEY7 | pwp2h | PWP2 periodic tryptophan protein homolog (yeast);pwp2h;ortholog | PERIODIC TRYPTOPHAN PROTEIN 2 HOMOLOG (PTHR19858:SF0) | small subunit biogenesis |
| DANRE\|ZFIN=ZDB-GENE-060518-1\|UniProtKB=A0A0R4IBJ2 | bxdc2 | Brix domain-containing 2;bxdc2;ortholog | RIBOSOME BIOGENESIS PROTEIN BRX1 HOMOLOG (PTHR13634:SF0) | ribosomal protein(PC00202), large subunit biogenesis |
| DANRE\|ZFIN=ZDB-GENE-030131-6986\|UniProtKB=F1R6L6 | ncl | Nucleolin;ncl;ortholog | NUCLEOLIN (PTHR23003:SF34) | ribonucleoprotein(PC00201) |
| DANRE\|ZFIN=ZDB-GENE-050522-127\|UniProtKB=Q503Z6 | abt1 | Activator of basal transcription 1;abt1;ortholog | ACTIVATOR OF BASAL TRANSCRIPTION 1 (PTHR12311:SF7) | small subunit biogenesis |
| DANRE\|ZFIN=ZDB-GENE-041114-104\|UniProtKB=A0A0R4IRJ1 | tbl3 | Transducin (beta)-like 3;tbl3;ortholog | TRANSDUCIN BETA-LIKE PROTEIN 3 (PTHR19854:SF15) | G-protein coupled receptor(PC00021), small subunit biogenesis |
| DANRE\|ZFIN=ZDB-GENE-070410-68\|UniProtKB=A3KNP4 | bud23 | BUD23, rRNA methyltransferase and ribosome maturation factor;bud23;ortholog | 18S RRNA (GUANINE-N(7))-METHYLTRANSFERASE-RELATED (PTHR12734:SF0) | Methyltransferase (PC00155) |
| DANRE\|ZFIN=ZDB-GENE-050320-149\|UniProtKB=Q5BLF0 | nmd3 | 60S ribosomal export protein NMD3;nmd3;ortholog | 60S RIBOSOMAL EXPORT PROTEIN NMD3 (PTHR12746:SF2) |  |
| DANRE\|ZFIN=ZDB-GENE-030131-371\|UniProtKB=Q6NX08 | wdr12 | Ribosome biogenesis protein wdr12;wdr12;ortholog | RIBOSOME BIOGENESIS PROTEIN WDR12 (PTHR19855:SF11) | large subunit biogenesis |
| DANRE\|ZFIN=ZDB-GENE-030131-6378\|UniProtKB=Q7SY48 | heatr1 | HEAT repeat-containing protein 1;heatr1;ortholog | HEAT REPEAT-CONTAINING PROTEIN 1 (PTHR13457:SF1) | Ribonucleoprotein (PC00201), small subunit biogenesis |
| DANRE\|ZFIN=ZDB-GENE-021220-2\|UniProtKB=Q8JGR2 | ddx54 | ATP-dependent RNA helicase;ddx54;ortholog | ATP-DEPENDENT RNA HELICASE DDX54 (PTHR24031:SF292) |  |
| DANRE\|ZFIN=ZDB-GENE-050417-142\|UniProtKB=F1QTS9 | mrto4 | Ribosome assembly factor mrt4;mrto4;ortholog | MRNA TURNOVER PROTEIN 4 HOMOLOG (PTHR45841:SF1) | large subunit biogenesis |
| DANRE\|ZFIN=ZDB-GENE-040718-474\|UniProtKB=Q6DI25 | imp4 | IMP4, U3 small Nucleolar ribonucleoprotein, homolog (Yeast);imp4;ortholog | U3 SMALL NUCLEOLAR RIBONUCLEOPROTEIN PROTEIN IMP4 (PTHR22734:SF2) | Ribonucleoprotein (PC00201) |
| DANRE\|ZFIN=ZDB-GENE-031030-13\|UniProtKB=Q6PFJ1 | ngdn | Neuroguidin;ngdn;ortholog | NEUROGUIDIN (PTHR13237:SF7) | small subunit biogenesis |
| DANRE\|ZFIN=ZDB-GENE-021213-1\|UniProtKB=Q6NV26 | sdad1 | Protein SDA1 homolog;sdad1;ortholog | PROTEIN SDA1 HOMOLOG (PTHR12730:SF0) | large subunit biogenesis |
| DANRE\|ZFIN=ZDB-GENE-030131-3762\|UniProtKB=E7FCX4 | tsr1 | TSR1, 20S rRNA accumulation, homolog (S. cerevisiae);tsr1;ortholog | PRE-RRNA-PROCESSING PROTEIN TSR1 HOMOLOG (PTHR12858:SF1) | small subunit biogenesis |
| DANRE\|ZFIN=ZDB-GENE-040426-764\|UniProtKB=Q802W4 | nol10 | Nucleolar protein 10;nol10;ortholog | NUCLEOLAR PROTEIN 10 (PTHR14927:SF0) | small subunit biogenesis |
| DANRE\|ZFIN=ZDB-GENE-030219-109\|UniProtKB=A0JMQ0 | bop1 | Ribosome biogenesis protein bop1;bop1;ortholog | RIBOSOME BIOGENESIS PROTEIN BOP1 (PTHR17605:SF0) | ribosomal protein (PC00202), large subunit biogenesis |
| DANRE\|ZFIN=ZDB-GENE-040426-1995\|UniProtKB=Q6TNW3 | abce1 | ATP-binding cassette, sub-family E (OABP), member 1;abce1;ortholog | ATP-BINDING CASSETTE SUB-FAMILY E MEMBER 1 (PTHR19248:SF16) |  |
| DANRE\|ZFIN=ZDB-GENE-021231-2\|UniProtKB=Q3KRG3 | tsr2 | Pre-rRNA-processing protein TSR2 homolog;tsr2;ortholog | PRE-RRNA-PROCESSING PROTEIN TSR2 HOMOLOG (PTHR21250:SF0) | small subunit biogenesis |
| DANRE\|ZFIN=ZDB-GENE-050417-27\|UniProtKB=F8W3K6 | exosc2 | Exosome component 2;exosc2;ortholog | EXOSOME COMPLEX COMPONENT RRP4 (PTHR21321:SF4) | Esterase (PC00097); exoribonuclease (PC00099) |
| DANRE\|ZFIN=ZDB-GENE-060623-1\|UniProtKB=A0A0R4IJV5 | ddx52 | DEAD (Asp-Glu-Ala-Asp) box polypeptide 52;ddx52;ortholog | ATP-DEPENDENT RNA HELICASE DDX52-RELATED (PTHR24031:SF594) | small subunit biogenesis |
| DANRE\|ZFIN=ZDB-GENE-030131-419\|UniProtKB=Q4V9P9 | nol11 | Nucleolar protein 11-like;nol11;ortholog | NUCLEOLAR PROTEIN 11 (PTHR15633:SF2) | small subunit biogenesis |
| DANRE\|ZFIN=ZDB-GENE-030131-533\|UniProtKB=Q6PBV6 | nhp2 | H/ACA ribonucleoprotein complex subunit 2-like protein;nhp2;ortholog | H/ACA RIBONUCLEOPROTEIN COMPLEX SUBUNIT 2 (PTHR23105:SF12) | ribosomal protein(PC00202), large subunit biogenesis |
| DANRE\|ZFIN=ZDB-GENE-990415-206\|UniProtKB=P79741 | pes | Pescadillo;pes;ortholog | PESCADILLO HOMOLOG (PTHR12221:SF6) | ribosomal protein(PC00202) |
| DANRE\|ZFIN=ZDB-GENE-060427-1\|UniProtKB=F1R3Q7 | rrp9 | Ribosomal RNA-processing 9, small subunit (SSU) processome component, homolog (yeast);rrp9;ortholog | U3 SMALL NUCLEOLAR RNA-INTERACTING PROTEIN 2 (PTHR19865:SF0) | large subunit biogenesis |
| DANRE\|ZFIN=ZDB-GENE-030131-9828\|UniProtKB=A0A0R4IQB8 | ftsj3 | pre-rRNA processing protein FTSJ3;ftsj3;ortholog | PRE-RRNA PROCESSING PROTEIN FTSJ3 (PTHR10920:SF13) | large subunit biogenesis |
| DANRE\|ZFIN=ZDB-GENE-040426-1543\|UniProtKB=A5WVQ0 | nat10 | RNA cytidine acetyltransferase;nat10;ortholog | RNA CYTIDINE ACETYLTRANSFERASE (PTHR10925:SF5) | small subunit biogenesis |
| DANRE\|ZFIN=ZDB-GENE-040426-1936\|UniProtKB=Q7ZTZ4 | fbl | Fibrillarin;fbl;ortholog | FIBRILLARIN (PTHR10335:SF0) | Methyltransferase (PC00155); ribonucleoprotein (PC00201) |
| DANRE\|ZFIN=ZDB-GENE-030131-3831\|UniProtKB=Q7ZW33 | utp15 | U3 small nucleolar RNA-associated protein 15 homolog;utp15;ortholog | U3 SMALL NUCLEOLAR RNA-ASSOCIATED PROTEIN 15 HOMOLOG (PTHR19924:SF26) |  |
| DANRE\|ZFIN=ZDB-GENE-040426-2317\|UniProtKB=A0A0R4IXF1 | gnl2 | Nucleolar GTP-binding protein 2;gnl2;ortholog | NUCLEOLAR GTP-BINDING PROTEIN 2 (PTHR11089:SF9) | large subunit biogenesis |
