# Supplementary material for: RNA exosome mutations in pontocerebellar hypoplasia alter ribosome biogenesis and p53 levels
Source: Life Sci Alliance. 2020 Jun 11;3(8):e202000678. doi: 10.26508/lsa.202000678 (PMC7295610; doi:10.26508/lsa.202000678)
Supplement: Supplementary file 5 [file LSA-2020-00678_SdataF5.pptx]

## Slide 1
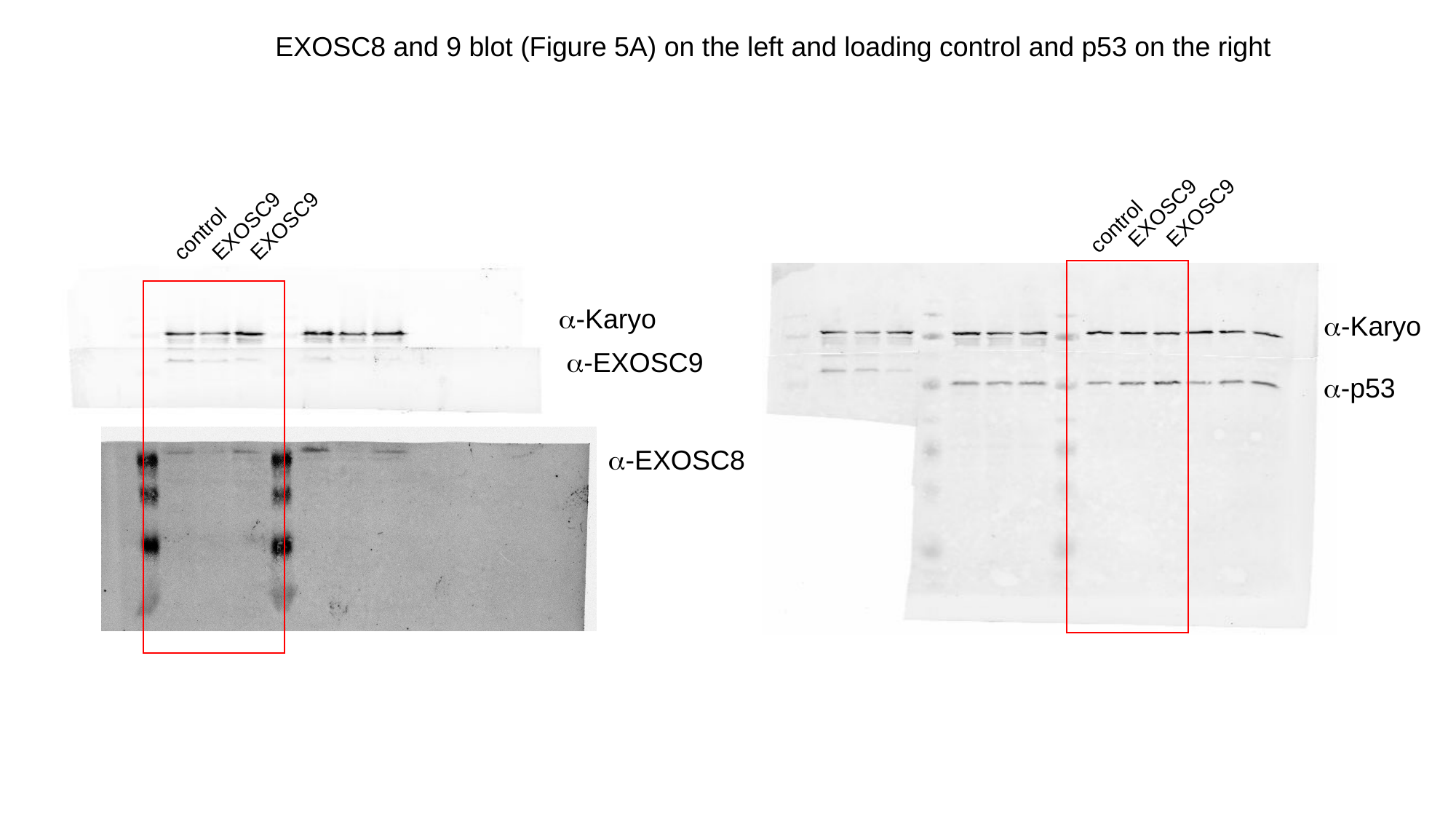

EXOSC8 and 9 blot (Figure 5A) on the left and loading control and p53 on the right
EXOSC9
EXOSC9
control
EXOSC9
control
EXOSC9
a-Karyo
a-Karyo
a-EXOSC9
a-p53
a-EXOSC8

## Slide 2
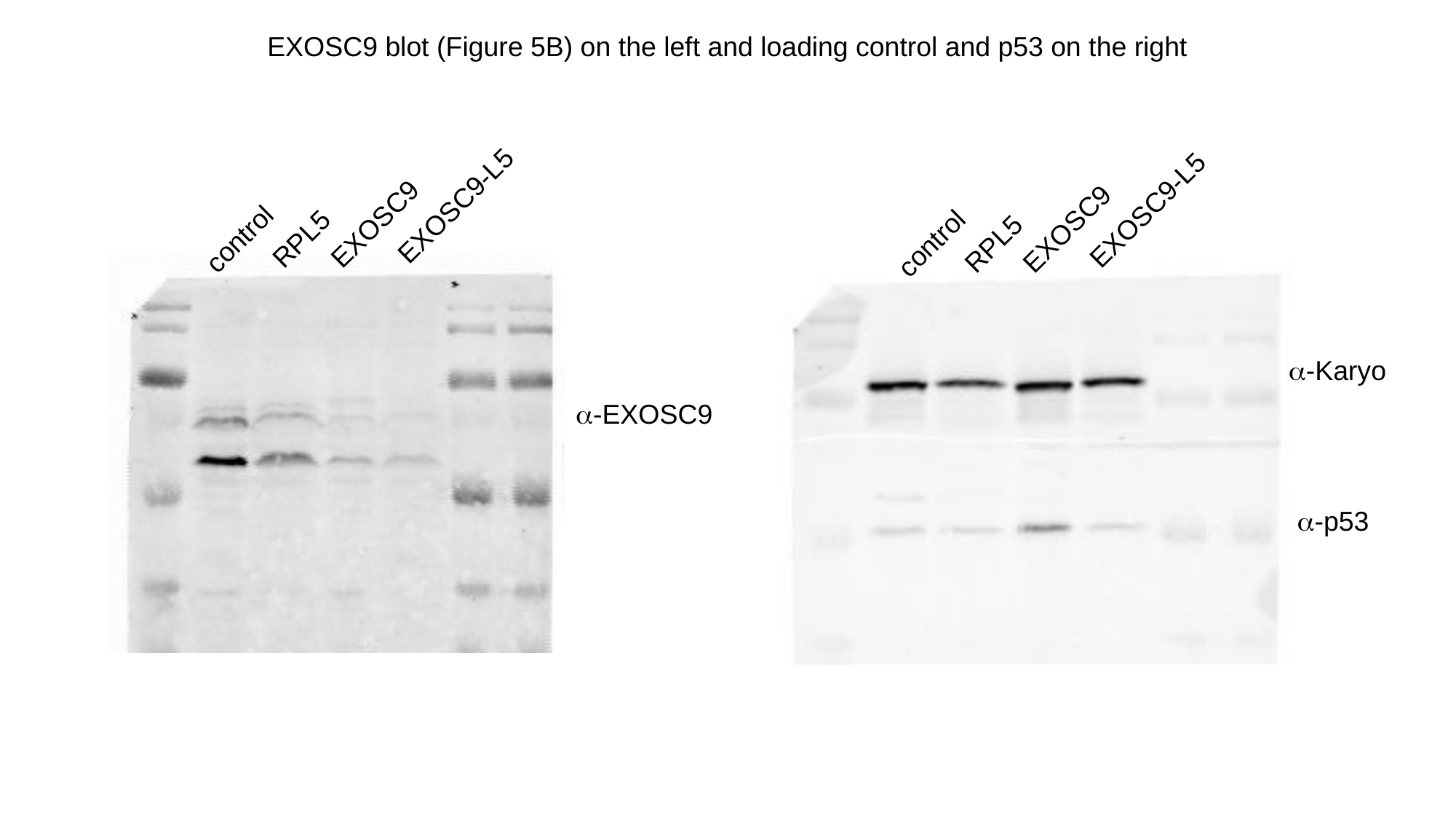

EXOSC9 blot (Figure 5B) on the left and loading control and p53 on the right
EXOSC9-L5
RPL5
EXOSC9-L5
EXOSC9
RPL5
control
EXOSC9
control
a-Karyo
a-EXOSC9
a-p53
