# Supplementary figures and images for: RNA exosome mutations in pontocerebellar hypoplasia alter ribosome biogenesis and p53 levels
Source: Life Sci Alliance. 2020 Jun 11;3(8):e202000678. doi: 10.26508/lsa.202000678 (PMC7295610; doi:10.26508/lsa.202000678)

**
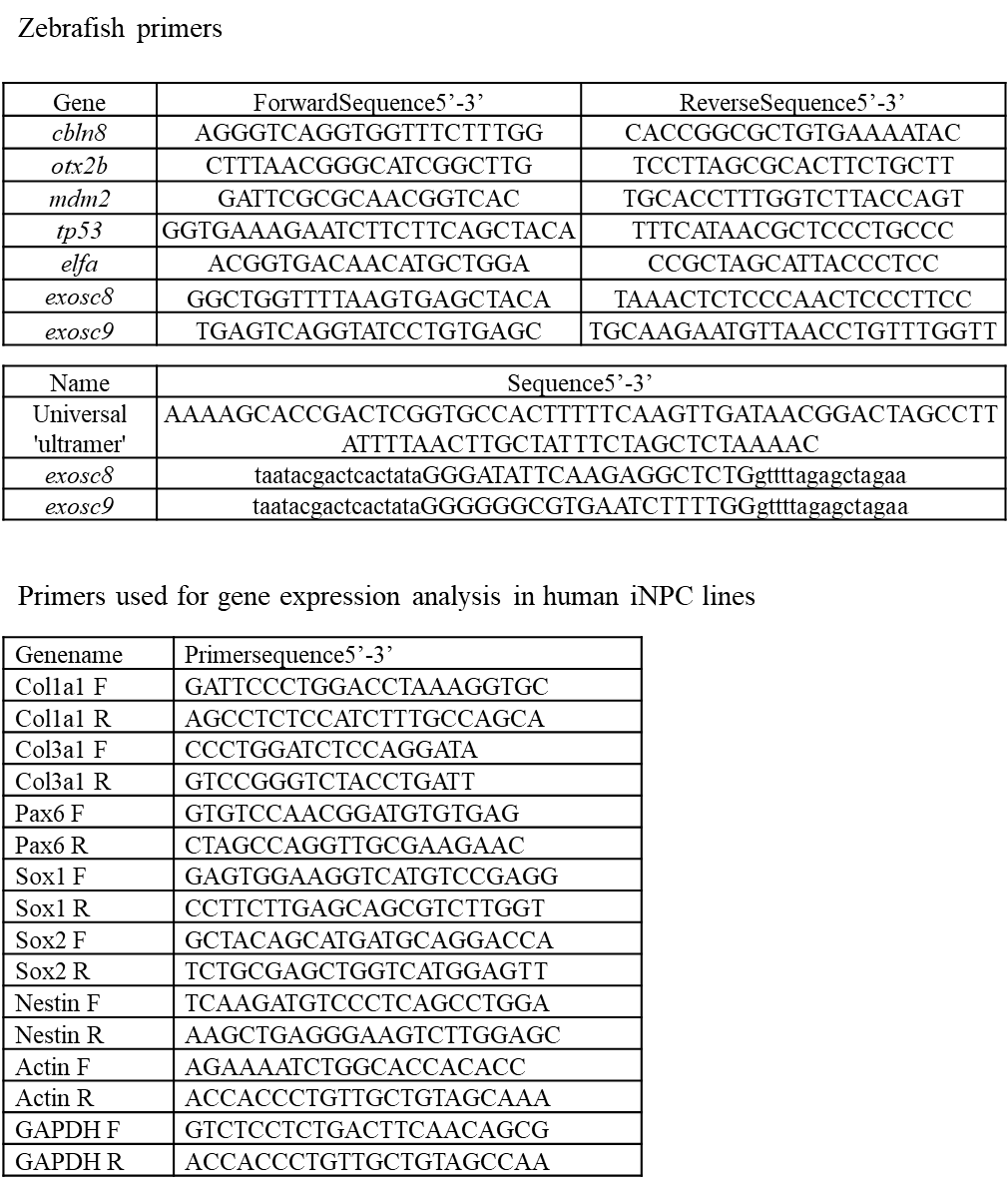
**

Supplement: Supplementary file 8 [file LSA-2020-00678_TableS7.docx]
